# Filgrastim therapy in a child with neutropenia induced by linezolid

**DOI:** 10.1007/s11096-013-9814-8

**Published:** 2013-07-03

**Authors:** Marta Hernández Segurado, Maria Ángeles Arias Moya, Marta Gómez Pérez, Macarena Bonilla Porras, Eva Castillo Bazán, Francisco Javier Bécares Martínez, Gema Toledano Mayoral, María Isabel Panadero Esteban

**Affiliations:** Pharmacy Service, Fundación Jiménez Díaz University Hospital, Avenue Reyes Católicos, 2, 28040 Madrid, Spain

**Keywords:** Filgrastim, Linezolid, Multidrug-resistant tuberculosis, Neutropenia

## Abstract

We describe the case of a young child with multidrug-resistant tuberculosis, treated with linezolid. The child developed severe neutropenia after 5 months of treatment. Filgrastim was used, a drug that officially is not indicated for non-cytostatic drug-induced neutropenia. This allowed the fast recovery of the patient’s neutrophil-count. However, more experience with the off-label use of filgrastrim is needed in the pediatric population.

## Impacts on practice

- The possible occurrence of myelosuppression secondary to treatment with linezolid is well known.
- There is no evidence of pharmacological management of these adverse effects caused by linezolid in adults, even in the pediatric population.
- The indication of filgrastim in the product information does not include non cytostatic drug-induced neutropenia.

## Introduction

Linezolid is a synthetic antibacterial agent belonging to the oxazolidinones group. It is active against Gram-positive bacteria by inhibiting protein synthesis by binding bacterial ribosome (23S rRNA inside the 50S subunits), preventing the formation of functional 70S initiation complex, which is an essential component of the translation process [1]. Linezolid is approved for the treatment of community-acquired or nosocomial pneumonia as well as for infections of skin and soft tissues. However, it has also been used for the treatment of multidrug-resistant tuberculosis in adults and pediatric population [2]. WHO classifies drugs for the treatment of multidrug-resistant tuberculosis infection (MDR-TB) in 5 groups, corresponding to the fifth group linezolid: Antituberculosis agents with currently unclear efficacy [3].

Regarding adverse reactions, cases of myelosuppression have been reported, including anemia, leukopenia, pancytopenia, and thrombocytopenia. In most cases the hematological parameters normalize after discontinuation of the linezolid treatment. The risk of these effects appears to be associated with the duration of the treatment. Monitoring hemogram is advised in patients who present the next clinical situations: prior anemia, granulocytopenia or thrombocytopenia; receiving concomitant medications that may alter the blood count; severe renal insufficiency; or receiving more than 10–14 days of linezolid treatment. In such patients, linezolid treatment is only advisable if close monitoring of hemoglobin levels, blood count and platelets is ensured [1].

Next we describe a case of neutropenia in a pediatric patient diagnosed with MDR-TB treated with linezolid, where the filgrastim rescue treatment was necessary.

## Case description

A 15 month old boy with a recent diagnose of tuberculosis was admitted to our hospital for isolation and treatment.

The patient had had frequent contact over the past months with his uncle (index case), diagnosed with MDR-TB. The child was found to have a negative Mantoux test (0 mm) 3 months ago. After periodic inspections and due to the patient’s age, the family was advised for emergency admission for a positive result on Quantiferon tests, Mantoux (12 mm) and gastric juice PCR for Mycobacteria.

On admission, the child was clinically asymptomatic. The patient had been vaccinated according to the Spanish schedule, which does not include the BCG vaccine. The chest computed tomography showed lymphadenopathy consistent with the diagnosis of mediastinic lymph node tuberculosis.

After these additional studies prior to treatment, the child began anti-tuberculosis therapy with: moxifloxacin 100 mg/day orally administrated, ethionamide 125 mg/12 h orally administrated, cycloserine 125 mg/12 h orally administrated, linezolid 60 mg/12 h orally administrated, pyridoxine 25 mg/day orally administrated, and capreomycin 175 mg/day by subcutaneous reservoir. The study of the last sample was performed in another hospital; the strain was sensitive to all these drugs except linezolid which was not studied. The child was kept in strict respiratory isolation until 15 days of treatment were completed. Analytical tests and electrocardiogram were performed weekly to monitor the adverse effects that might appear as a result of the treatment. One month later the patient was discharged, continuing outpatient treatment that was well tolerated without adverse effects.

In the two subsequent analytical, the child showed moderate neutropenia, with an absolute neutrophil count (ANC) of 840 and 600/μl, respectively. The patient recovered spontaneously on both occasions, without modification or discontinuation of linezolid. The fluctuating course of neutropenia, even when linezolid was continued, was attributed by doctors to a recurrent virus infection.

**Fig. 1:**
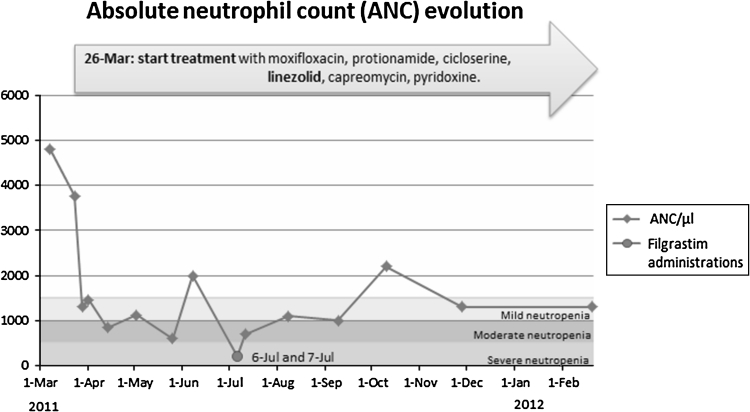
Patient’s treatment and ANC evolution from the beginning of therapy

Two months after, severe neutropenia was detected in the patient with an ANC of 200/μl. They finally decided to treat the patient with granulocyte colony-stimulating factor, filgrastim, due to the fact that the number of neutrophils was <500/μl. The child received only two subcutaneous doses of 65 mcg of filgrastim (5 mcg/kg), after which he gradually recovered in neutrophils count. Patient’s ANC evolution from the beginning of treatment is shown in Fig. 1. The child has remained with mild neutropenia until today, without additional doses of filgrastim or suspending nor modifying the treatment with linezolid.

## Discussion

The MDR-TB is defined as in vitro resistance to isoniazid and rifampicin [3]. Young children (<5 years) are at high risk for developing disease after infection, being predisposed to a new (not previously treated) MDR-TB [4]. Exposure, pretreatment and drug sensitivity should be considered for each patient [5]. The optimal number of drugs needed for the successful treatment of drug-resistant tuberculosis infection (DR-TB) is unclear because of the lack of controlled trials comparing different regimens. Most guidelines are based on expert opinions or uncontrolled case series from a single institution. The guidelines for adults recommend regimens of at least 4 drugs including at least 3 previously unused drugs, of which one should be an injectable agent, and at least 2 with bactericidal activity. Regimens should start with Group 1 drugs: Isoniazid, rifampin, ethambutol, pyrazinamide; moving on to the next group only when no adequate drug is left in the previous group. Most experts suggest that 1 drug should be selected from both group 2 (parenteral aminoglycosides or tuberactinomycin) and 3 (fluoroquinolones) agents, because of higher documented cure rates in regimens including these drugs. There are two other groups of drugs active against MDR-TB, the fourth group would be made up of second-line drugs like ethionamide; prothionamide; cycloserine; terizidone, p-aminosalicylic acid; thioacetazone. The fifth group would be formed by antituberculosis agents with unclear efficacy like clofazimine; amoxicillin/clavulanate; clarithromycin; azithromycin; linezolid; imipenem/cilastin [3]. Our patient received drugs from the five groups. Regimens in which drugs are administered only twice or thrice weekly are not recommended for DR-TB. In pediatric patients, treatment duration depends on the extent and context of the DR-TB disease, but in most cases should be 12–18 months from the first negative culture [3].

Linezolid has activity in vitro and in vivo against MDR-TB [4]. The recommended dose in pediatric patients is 10 mg/kg/12 h. Myelosuppression caused by linezolid is widely described, as well as the incidence of neutropenia, which in pediatric population has been registered between 1.2 and 6.4 % [6].

The reported experience of the use of linezolid in pediatric patients for the treatment of MDR-TB is quite high. However, published by the management of adverse events caused by the drug in this population, particularly for neutropenia, is limited to a case series report, in which three patients experienced reversible neutropenic episodes without pharmacological management, in addition to another adverse effects caused by linezolid [7]. Safety data sheets indicate that linezolid-induced neutropenia is reversible in most cases when treatment is stopped, but in the case of severe neutropenia should take the necessary therapeutic measures. Also, the neutropenia is more pronounced in patients whose treatment exceeds 28 days (when it is used off-label) [1]. In this case the discontinuation of linezolid treatment was not considered because of the proved efficacy of this drug in the MDR-TB.

Filgrastim, a methionyl-stimulator factor of the human granulocyte colony, is obtained by recombinant DNA technology in *E. coli* (K12). It is indicated to reduce the duration of neutropenia and the incidence of febrile neutropenia (ANC ≤ 500/μl) in patients treated with cytotoxic chemotherapy or who have severe congenital neutropenia, cyclic, or serious idiopathic; as well as in the treatment of persistent neutropenia in patients with advanced HIV infection to reduce the risk to develop bacterial infections when other options to manage neutropenia are not appropriate [8].

Patients with neutropenia caused by non-cytostatic drugs don’t have the same risk of viral, fungal and parasitic infections as the patient receiving chemotherapy treatment. However, for those children who do not routinely receive filgrastim, its use may be appropriate [9]: data exist on the use of granulocyte colony-stimulating factors to increase the ANC and reduce episodes of fever and infection in pediatric patients with different types of neutropenia [10].

The indication of filgrastim in the product information does not include non-cytostatic drug-induced neutropenia; for this reason, in the absence of alternatives, treatment was processed in accordance with Royal Decree 1015/2009 of June 19th, which regulates the availability of drugs in special situations (off-label Spanish legislation).

The safety and efficiency of filgrastim are similar in adults and children in that indications [8]. According to that, the handling of severe neutropenia of this child was extrapolated from the treatment of pediatric patients with chemotherapy-induced neutropenia.

## Conclusion

The use of filgrastim in severe neutropenia induced by other drugs has been beneficial in this case and can serve as a guide in other patients where the drug causing severe neutropenia cannot be withdrawn.

As there is no data in the literature, further studies would be needed to make recommendations about the dose and duration of treatment with filgrastim for non-chemotherapy drug-induced severe neutropenia, especially in the pediatric population due to differences in pharmacokinetics, pharmacodynamics and adverse reactions compared to adults. In this case we use the indicated dose used in patients receiving chemotherapy, (5 mcg/kg) for 2 days, because after that time the number of neutrophils recovered and no more doses of filgrastim were needed.

In addition, as it was shown, a close monitoring should be realized of laboratory parameters of patients treated with linezolid, both pediatric and adults, because of severe hematologic adverse effects it can produce.
